# Ras inhibition enhances autophagy, which partially protects cells from death

**DOI:** 10.18632/oncotarget.703

**Published:** 2013-01-19

**Authors:** Eran Schmukler, Efrat Grinboim, Sari Schokoroy, Adva Amir, Eya Wolfson, Yoel Kloog, Ronit Pinkas-Kramarski

**Affiliations:** ^1^ Department of Neurobiology, Tel-Aviv University, Ramat-Aviv, Israel

**Keywords:** autophagy, Ras, transformation, signal transduction

## Abstract

Autophagy, a process of regulated turnover of cellular constituents, is essential for normal growth control but may be defective under pathological conditions. The Ras/PI3K/mTOR signaling pathway negatively regulates autophagy. Ras signaling has been documented in a large number of human cancers. In this *in-vitro* study we examined the effect of the Ras inhibitor Salirasib (S-*trans*, *trans*-farnesylthiosalicylic acid; FTS) on autophagy induction and cell viability. We show that Ras inhibition by FTS induced autophagy in several cell lines, including mouse embryonic fibroblasts and the human cancer cell lines HeLa, HCT-116 and DLD-1. The autophagy induced by FTS seems to inhibit the cell death induced by FTS, since in the absence of autophagy the death of FTS-treated cells was enhanced. Therefore, inhibition of autophagy may promote the inhibition of tumor cell growth and the cell death mediated by FTS.

## INTRODUCTION

Autophagy is a process of self-digestion of cellular constituents through an autophagosomic-lysosomal pathway [1, 2]. It is important for normal growth control but may be defective under pathological conditions [3, 4]. Autophagy plays an essential role in maintaining the balance between the formation and the degradation of proteins and as a cell-survival mechanism under stressful conditions such as absence of nutrients. Thus, autophagy is important for normal cell growth, differentiation and survival [5]. Autophagy has been linked to some forms of cancer, including pancreatic and breast carcinomas and hepatoma [4]. Studies on regulation of autophagy by the autophagy-regulating genes *Atg* (*Apg*/*Aut*) have contributed to our understanding of the molecular control of autophagy [1, 6]. Formation of the autophagosome requires class III phosphatidylinositol 3-kinase (Vps34) [7], which forms a complex with the Atg6 (Beclin 1) protein [8]. Autophagy can be also negatively regulated by the mTOR signaling pathway, which is regulated by Ras [9].

The Ras family of small GTPases transmit extracellular signals, which are initiated by cell-surface receptors and serve to regulate diverse cellular processes including the growth, differentiation, motility and death of cells [10]. Signals transmitted by activated Ras are mediated through the protein's interaction with multiple effectors including mitogen-activated kinase (MAPK), phosphoinositide-3-kinase (PI3K) and Ral-GEF [10, 11]. Ras signaling is activated in a large number of human cancers [12]. Mutations of codons 12, 13 and 61 in *Ras* result in constitutively active Ras, and activating mutations of the three major Ras isoforms (H, K and N) have been found in more than 30% of human cancers [13-15].

Because Ras signaling represents a convergence juncture for many different extracellular signals, Ras and its effectors may be appropriate targets for therapeutic intervention. Ras is post-translationally modified by the addition of a farnesyl lipid group that allows its attachment to the cell membrane [16]. Attempts have therefore been made to block Ras or Ras-dependent functions in cancer cell lines by the use of farnesyltransferase inhibitors [17-19]. S-*trans, trans-*farnesylthiosalicylic acid (FTS; also known as Salirasib) is a synthetic Ras inhibitor that structurally resembles the carboxy-terminal farnesylcysteine group common to all Ras proteins. FTS acts as a functional Ras antagonist in cells, affecting Ras/membrane interactions, dislodging the protein from its anchorage domains and facilitating its degradation, thereby reducing cellular Ras content [20, 21]. FTS inhibits the growth of H-Ras-, K-Ras- and N-Ras-transformed rodent fibroblasts *in vitro* [22, 23]. In addition, FTS can inhibit the anchorage-dependent growth of LNCaP, PC3 and CWR-R1 cells [24, 25]. Furthermore, FTS inhibits growth and induces apoptosis of cancer cell lines such as hepatocarcinoma and prostate cancer [25, 26]. In a number of cancers, however, tumor cells do not undergo apoptosis when treated with FTS. These include pancreatic [27], colon [28, 29] and lung cancer cell lines that express mutant K-Ras [30], an important target for FTS.

In this *in-vitro* study we examined the impact of FTS on autophagy and cell growth, in mouse embryonic fibroblsts (MEFs) and in various human cancer cell lines, and determined the contribution of autophagy to cell viability in response to FTS treatment. Our results demonstrated that FTS both induces autophagy and inhibits cell growth. They further showed that inhibition of autophagy promotes FTS-induced cell death and inhibition of cell growth.

## RESULTS AND DISCUSSION

Recent studies suggest that inhibition of autophagy may become a new strategy for cancer therapy. Those studies demonstrated that some cancers depend on autophagy for survival during external stresses such as hypoxia, chemotherapy or radiotherapy [31]. Other studies have suggested the possible involvement of both Ras and autophagy in cancer cell transformation [32, 33]. It was not known, however, whether inhibition of Ras by small molecules can affect autophagy. The present study was aimed at determining the effect of Ras inhibition by FTS (Salirasib) on autophagy and on cell viability.

**Figure 1:**
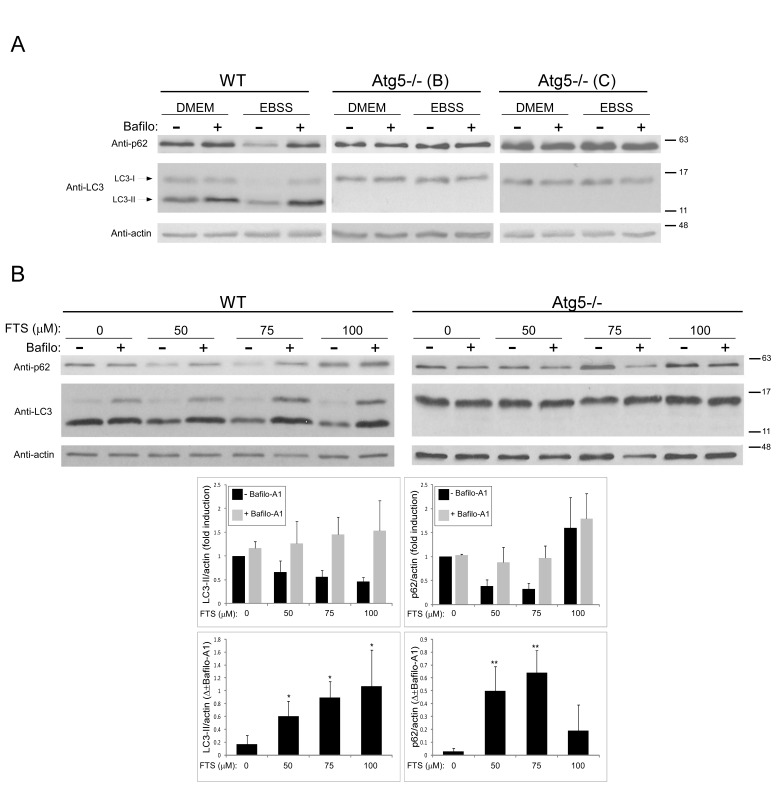
FTS induces autophagy in wild-type MEFs but not in Atg5^−/−^ MEFs (A) WT and Atg5^−/−^ (clones B and C) MEFs were cultured in complete DMEM or in EBSS with or without the autophagy inhibitor bafilomycin A^−/−^ (10 nM) for 3 hours, and were then subjected to immunoblot analysis using anti-LC3 or anti-p62 Abs. Blots were reacted with anti-actin Abs as loading control. (B) WT and Atg5^−/−^ MEFs were treated with FTS at the indicated concentrations with or without 10 nM bafilomycin A^−/−^ for 24 hours and were then subjected to immunoblot analysis using anti-LC3, anti-p62 or anti-actin Abs. Upper panel: Representative blots are shown. Lower panel: Densitometric analysis of WT MEF results at different concentrations of FTS, presented as fold induction (with or without bafilomycin A^−/−^) over the control untreated cells (upper panels) and as the difference between measured values (with or without 10 nM bafilomycin A^−/−^) (lower panels). *, p < 0.05 and **, p<0.01 compared to untreated cells; n=3. Values are means ± S.D.

For assessment of autophagy, we used LC3 protein as a marker. When autophagy is induced this protein undergoes lipidation, and the lipidated LC3 (LC3-II) marks the autophagosomal membrane [6]. LC3 levels were determined in wild-type (WT) mouse embryonic fibroblasts (MEFs) and in Atg5^−/−^ MEFs that do not undergo autophagy because Atg5 is required both for autophagy and for LC3-II formation [34]. First, we verified the inability of Atg5^−/−^ MEFs to undergo autophagy under standard autophagy-inducing conditions. Cells were cultured under normal conditions (in DMEM) or under conditions of nutrient (amino-acid) deprivation (in EBSS). Figure 1A shows that lysates of WT MEFs contain both LC3-I (the non-lipidated form) and LC3-II proteins, whereas Atg5^−/−^ MEF lysates express only LC3-I. Under nutrient deprivation, WT MEFs exhibited enhanced autophagy flux as reflected by the marked decrease in LC3-II. This apparent consumption of LC3-II could be inhibited by bafilomycin A_1_ (a specific inhibitor of vacuolar type H+-ATPase (V-ATPase) that inhibits fusion of autophagosomes with the lysosome, thereby blocking autophagy). In Atg5^−/−^ MEFs, however, no change in LC3-I levels was observed under the same conditions and no LC3-II protein was observed. We also examined the expression level of p62/SQSTM1, a protein that binds to LC3 and is degraded by autophagy [35]. As shown, under the same conditions, p62 was reduced in WT MEFs but not in Atg5^−/−^ MEFs. Taken together these results strongly suggest that Atg5^−/−^MEFs indeed cannot undergo autophagy.

Next we examined whether autophagy can be induced by FTS. To measure autophagic flux, cells were treated with FTS in the presence or absence of bafilomycin A_1._ As shown in Figure 1B, in WT MEFs, in the absence of bafilomycin A_1_, LC3 levels decreased (reflecting enhanced autophagy), but LC3-II was increased upon addition of the inhibitor. These findings suggest that the FTS treatment induced autophagy in WT MEFs. Figure 1B shows, however, that in Atg5^−/−^ MEFs (which are constitutionally incapable of autophagy), LC3 levels were unaffected by treatment with FTS. To further study the FTS-induced autophagy, we examined the expression levels of p62/SQSTM1 in the FTS-treated cells. FTS at 50‒75μM enhanced p62 degradation in the WT MEFs, presumably due to autophagy. At 100 μM, however, FTS induced an increase in p62 possibly as a result of p62 synthesis. At this concentration, furthermore, there was no significant difference between bafilomycin A_1_-treated and -untreated cells.

**Figure 2:**
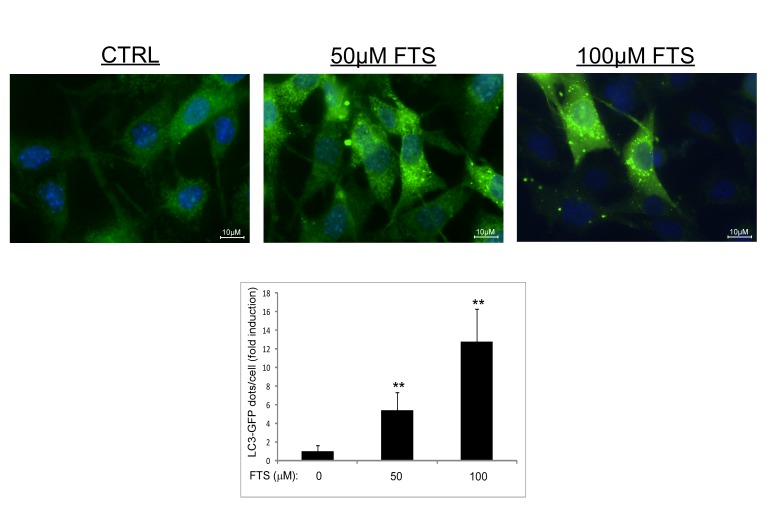
LC3-GFP expression in MEFs MEFs stably expressing LC3-GFP were treated with 50 and 100 μM FTS for 24 hours. The cells were fixed with 4% paraformaldehyde and nuclei were stained with bisbenzimide (Hoecsht 33258). Following fixation and staining, the cells were photographed using a Nikon optical fluorescence microscope Model TE-2000S (60×magnification). *Upper panel*: Representative images. *Lower panel*: Autophagy was quantified by counting the numbers of LC3 dots per cell using ImageJ software. Values are representative results (means ± S.D.) of two independent experiments, in each of which 60‒100 cells were analyzed per treatment. **, p < 0.01.

To further demonstrate autophagy induction, we repeated the above experiment using WT MEFs stably expressing GFP-LC3. As shown in Figure 2, FTS induced enhanced autophagosome formation as reflected by enhanced punctated staining of GFP-LC3. These findings further support our results suggesting that FTS can induce autophagy in MEFs.

**Figure 3:**
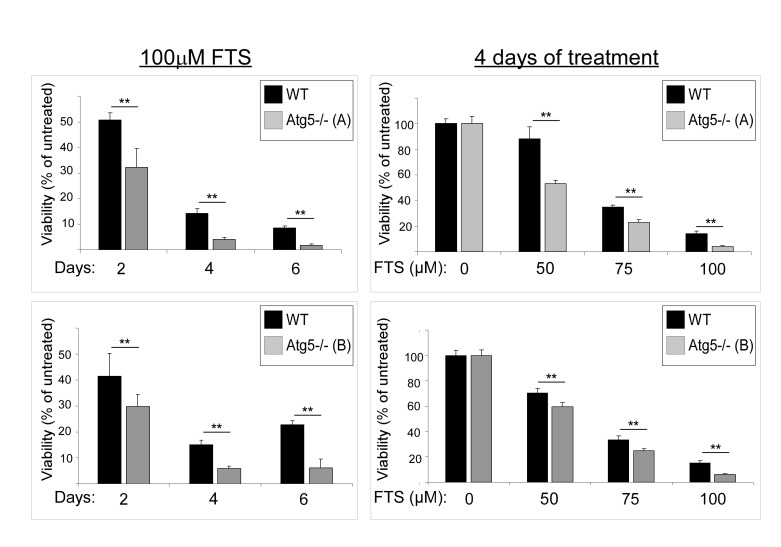
FTS treatment reduces numbers of Atg5^−/−^ MEFs more effectively than of wild-type MEFs WT and Atg5^−/−^ MEFs (clones B and C, upper and lower graphs, respectively) were treated with 100 μM FTS for the indicated time periods (left panels) and for 4 days at the indicated concentrations (right panels), and were then tested for cell viability using the methylene blue staining assay. Results are presented as percent of untreated control cells. Values are means ± S.D of 4‒6 determinations. **, p < 0.001.

**Figure 4:**
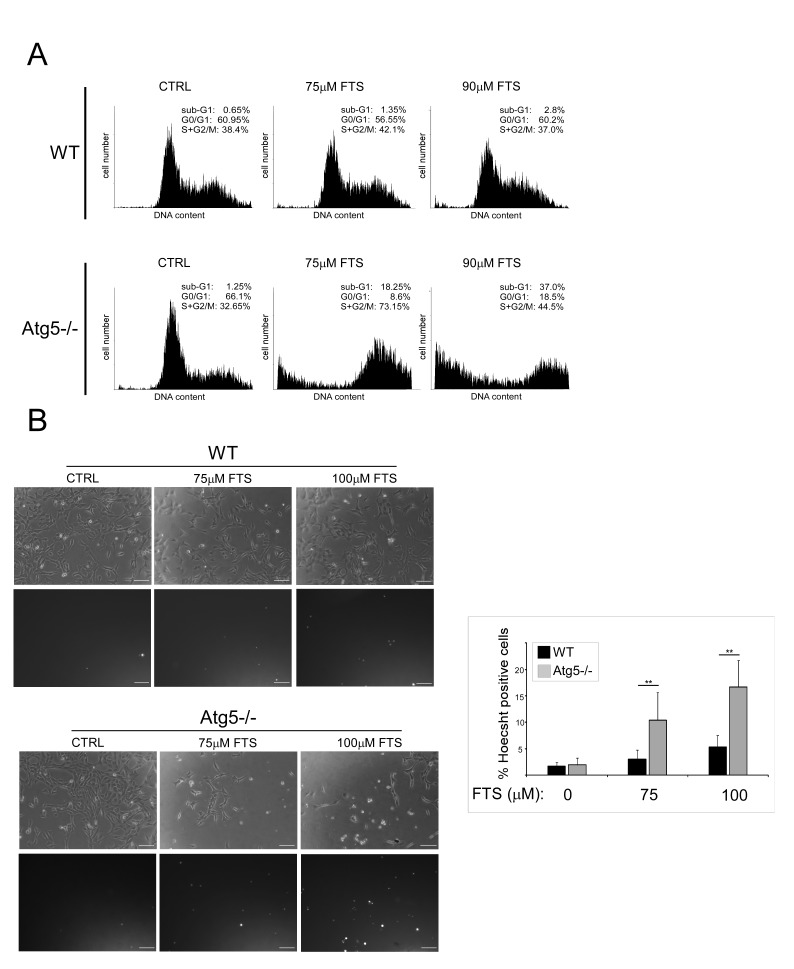
FTS induces death of Atg5^−/−^ MEFs more effectively than of wild-type MEFs (A) WT and Atg5^−/−^ MEFs were treated for 3 days with FTS (75 μM or 90 μM). The cells were then harvested and analyzed for their DNA content by flow cytometry. The percentage of live cells at different cell cycle stages is indicated. (B) WT and Atg5^−/−^ MEFs were treated for 4 days with FTS (75 μM or 100 μM). The treated cells were stained with the fluorescent DNA dye bisbenzimide (Hoechst 33258, 1 μg/ml) to determine the number of dead cells. After staining the cells were photographed with an Olympus optical inverted phase-contrast microscope Model IX70 (20× magnification; scale bars, 100 μm). *Left:* Representative images. *Right:* In each field (10‒15 fields for each treatment) the percentage of dying cells was estimated by counting the Hoechst-positive cells after each treatment, subtracting this number from the total number of cells (100‒200 cells per field), and expressing the result as a percentage of the total cell number. Values are presented as means ± S.D. **, p < 0.001.

We next examined whether FTS treatment affects cell viability and, if so, whether the cell viability is affected by autophagy induction. For these experiments we again used WT MEFs and Atg5^−/−^ MEFs. Cells were treated with FTS at the indicated time periods or concentrations, and cell viability was determined using the methylene blue staining assay. As shown in Figure 3, FTS treatment significantly inhibited cell viability but its effect was significantly more pronounced in the Atg5^−/−^ MEFs (examined in two different clones) than in the WT MEFs. These results suggest that although FTS inhibited cell viability, autophagy induced by the FTS treatment may have provided the cells with partial protection from such inhibition. This conclusion is supported by the results of two additional methods for detection of cell death, namely flow cytometry and Hoechst dye exclusion assay (Figure 4). FTS-induced cell death in Atg5^−/−^ MEFs was significantly more enhanced than in WT MEFs, as indicated both by the increase in the sub-G1 population (Figure 4A) and by the high percentage of Hoechst-positive cells (Figure 4B). Hence, our findings strongly suggest that FTS treatment of WT MEFs induces autophagy, which partially protects these cells from FTS-induced cell death and inhibition of cell growth.

**Figure 5:**
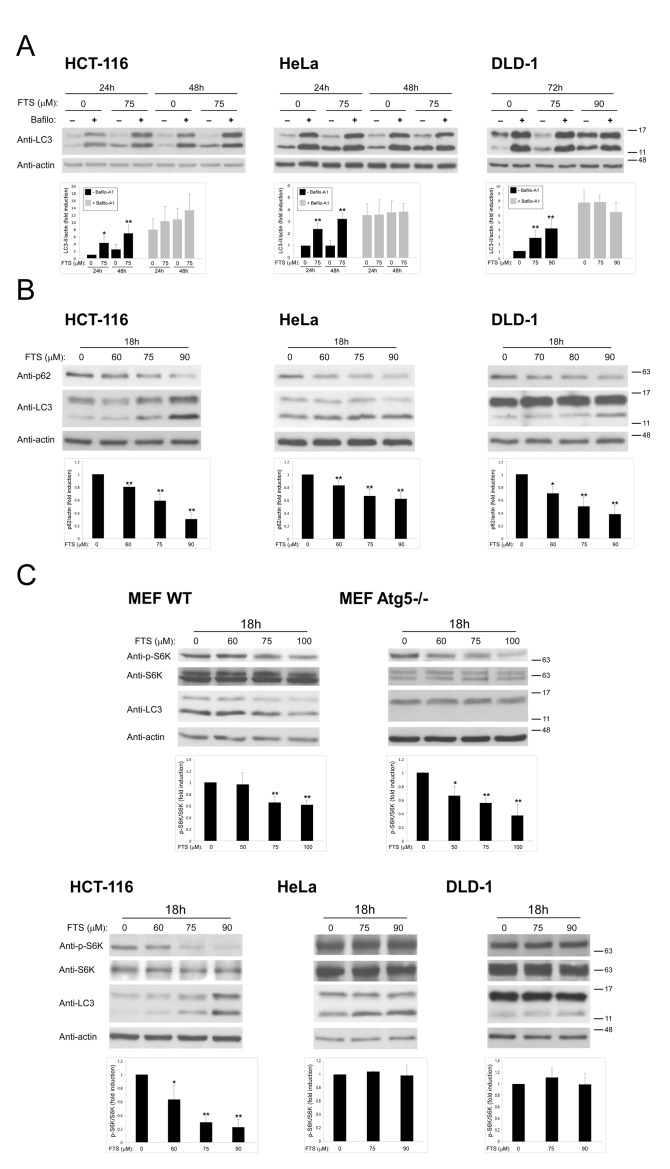
FTS induces autophagy in cancer cell lines (A) HCT-116, HeLa and DLD-1 cell lines were treated for the indicated times with FTS (75 μM or 90 μM), with or without 10 nM bafilomycin A_1_ The treated cells were subjected to immunoblot analysis using anti-LC3 Abs. (B, C) HCT-116, HeLa, DLD-1 and MEF (WT and Atg5−/−) cells were treated for 18 h with FTS at the indicated concentrations and were then subjected to immunoblot analysis using anti-LC3, anti-p62, anti-phospho-S6K and anti-S6K Abs. *Upper panel*s: Representative blots. *Lower panel*s: Densitometric analysis of the results, presented as fold induction over induction assessed in control untreated cells. *, p < 0.05 and **, p<0.01; n=3. Values are means ± S.D.

**Figure 6:**
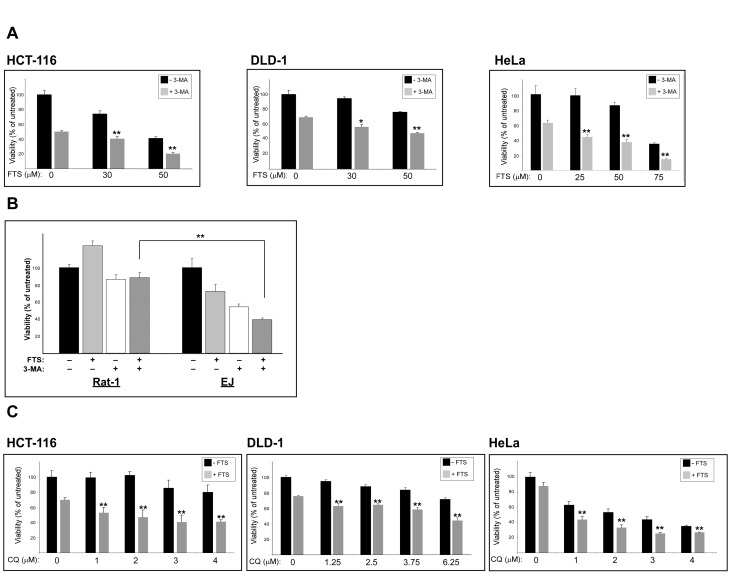
3-methyladenine and chloroquine, enhance FTS-induced inhibition of cell growth (A) HCT-116, HeLa, DLD-1 cells, were treated for 4 days with FTS at the indicated concentrations, with or without the addition of 10 mM 3-MA for the last 24 h. (B) EJ and Rat-1 cells were treated for 4 days with 90μM FTS, with or without the addition of 10 mM 3-MA for the last 24 h. The cells were then tested for cell viability using the methylene blue staining assay. Results are presented as percent of control; values are means ± S.D. of 4‒6 determinations. **, p < 0.01. (C) HCT-116, HeLa and DLD-1 cells were treated with 75 μM FTS with or without the addition of chloroquine (CQ) at the indicated concentrations for 4 days (DLD-1) or 5 days (HCT-116 and HeLa). The cells were then tested for cell viability using the methylene blue staining assay. Results are presented as percent of control; values are means ± S.D. of 4‒6 determinations. **, p < 0.01.

**Figure 7:**
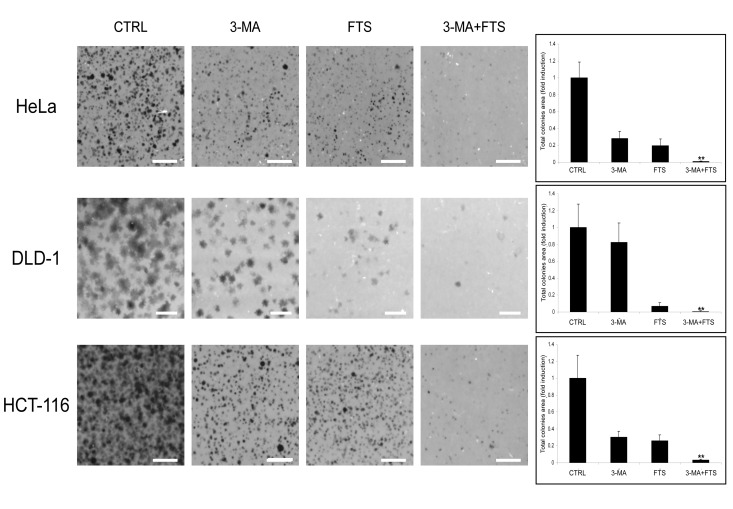
3-methyladenine enhances FTS-induced inhibition of cancer cell colonies formation (A) HCT-116, DLD-1 and HeLa cells were treated for 4 days with FTS (50, 63 and 75 μM, respectively) with or without the addition of 10 mM 3-MA for the last 24 h. The cells were then detached and recultured for another 5-10 days. Colonies were stained and the total colonies area was calculated as described in materials and buffers. Results are presented as percent of control; values are means ± S.D. of 10 determinations. **, p < 0.001 (scale bars, 5 mm).

Our findings of FTS-induced autophagy together with inhibition of cell growth prompted us to investigate whether these two effects of FTS are related and might be of relevance to cancer cells. The cells we used for this purpose were human cervical cancer (HeLa) and human colon cancer (HCT-116 and DLD-1) cell lines. First, we examined whether the observed effect of FTS on autophagy induction is specific to MEFs or is also applicable to other cells such as the above cancer cell lines. As shown in Figure 5, FTS treatment induced autophagy as judged by the enhanced conversion of LC3-I to LC3-II. Moreover, p62 levels were decreased by treatment with FTS compared to the control untreated cells (Figure 5B). These results further confirmed the ability of FTS treatment to induce autophagy. We also examined the effect of FTS treatment on S-6-kinase (S6K) phosphorylation (Figure 5C). Phosphorylated S6K was reduced in MEF and HCT-116 cells but not in HeLa and DLD-1 cells. Thus, only in certain cell types, FTS induced autophagy may depend on mTOR inhibition. Next, we inhibited autophagy induction using 3-methyladenine and chloroquine and examined whether this would affect the response of the cells to FTS treatment. As shown in Figure 6, cell viability in response to FTS was significantly lower in the presence than in the absence of 3-methyladenine or chloroquine. Of note, the effect of FTS and 3- methyladenine treatment on Rat-1 fibroblasts viability was significantly lower compared to the effect of the treatment on Ras transformed Rat-1 fibroblasts (EJ). To further examine the effect of Ras and autophagy inhibition on cell viability, we have used the long-term clonogenic assay (Figure 7). As shown, the combined treatment significantly reduced the number of colonies. Taken together, these results further supported the notion that autophagy, induced by FTS, partially protects cancer cells.

The precise role of autophagy in cancer depends on the cellular context of the cells. A large number of studies, summarized in a recent review [36], testify to the fact that autophagy can be both tumorigenic and tumor suppressive. Moreover, oncogenic Ras has been shown to be capable of elevating the pro-survival activity of autophagy [32, 37, 38]. Other studies have shown, however, that Ras-driven autophagy might be part of a pro-death mechanism. In particular, H-Ras-induced autophagy was recently shown to contribute to caspase-independent cell death [39]. It was also demonstrated that Ras upregulates Beclin 1 and that knockdown of the key autophagy genes *Beclin 1*, *Atg5* or *Atg7* reduces oncogenic Ras-mediated cell death [39, 40]. The results of the present study show that the Ras inhibitor FTS promoted autophagy in several cell lines. Our results also point to a pro-survival function of autophagy, as shown by the observation that in cells with impaired autophagy the FTS-induced cell death and inhibition of cell growth were significantly enhanced, and is further supported by confirmation of these results in three cancer cell lines. In these cells, both FTS-induced autophagy and inhibition of autophagy by the PI3K inhibitor, 3-methyladenine, enhanced FTS-mediated cell death. We suggest that FTS, in addition to inhibiting proliferation of cancer cells, induces pro-survival autophagy. Thus, inhibition of both Ras and autophagy may have a better effect on inhibition of cancer cell growth than inhibition of Ras or autophagy alone.

## MATERIALS AND METHODS

### Materials and buffers

The antibodies used were monoclonal mouse anti-actin (691001; MP Biomedicals, Santa Ana, CA), polyclonal rabbit anti-LC3B (L7543; Sigma-Aldrich, St. Louis, MO), polyclonal rabbit anti-phospho-Thr389-S6 kinase (S6311; Sigma-Aldrich), polyclonal rabbit anti-S6 kinase (S4047; Sigma-Aldrich) and polyclonal rabbit anti p62 (PM045; MBL Intenational, Woburn, MA). Salirasib (FTS, S-*trans, trans*-farnesylthiosalicylic acid), 3-methyladenine (3-MA; M9281), chloroquine (CQ, C6628) and bafilomycin A_1_ (B1793) were from Sigma.

### Cell lines

Mouse embryonic fibroblasts (MEFs) were grown in DMEM or EBSS (Gibco/Life Technologies, Bethesda, MD). The human cancer cell lines DLD-1, HCT-116 and HeLa were grown in RPMI-1640 (Gibco), McCoy's 5A modified medium (Sigma-Aldrich) and DMEM, respectively. Rat-1 fibroblast cells and H-Ras-transformed Rat-1 cells (EJ cells) were grown in DMEM. All media were supplemented with antibiotics and 10% heat-inactivated fetal bovine serum (FBS; Hyclone, Thermo Scientific, CITY, STATE). Cells were incubated at 37°C in 5% CO_2_ in air, and the medium was changed every 3‒4 days. When 70% confluent, cells were passaged in trypsin/disodium ethylenediaminetetraacetic acid (Biological Industries, Beit-Haemek, Israel). One day before treatment the cells were plated at ~50% confluence in medium supplemented with 5% fetal calf serum (10% for EJ and Rat-1 cells). Concentrations for FTS treatments (and their control treatments with 0.1% DMSO), as well as the duration of treatment where relevant, are indicated for each experiment.

### Stable transfections

MEFs were stably transfected with the Lipofectamine reagent (Invitrogen, Carlsbad, CA) according to the manufacturer's instructions. Stable clones expressing LC3-GFP were selected and cultured with 500 μg/ml geneticin (G-418, Calbiochem, San Diego, CA).

### Assays of cell survival and cell death

Cells were plated in medium supplemented with 5% FBS and treated as indicated for the different experiments. Cell numbers were determined by the methylene blue assay. For this purpose, the cells were fixed with 4% formaldehyde in phosphate-buffered saline for 2 hours, then washed once with 0.1 M boric acid (pH 8.5) and incubated with the DNA-binding dye methylene blue (1% in boric acid) for 20 minutes at room temperature. The cells were then washed three times and lysed with 0.1 M HCl. Absorbance was measured with a Tecan Spectrafluor Plus spectrophotometer (Mannedorf, Switzerland) at 595 nm. Cell viability was calculated as the ratio of absorbance in treated cultures to that in untreated control cultures. Dead cells were scored by nuclear staining and nuclear morphology. To estimate the number of dying cells, live cells were incubated for 10 minutes with 1 μg/ml of the fluorescent DNA dye bisbenzimide (Hoechst 33258; Sigma). After staining, the cells were photographed with an Olympus optical inverted phase-contrast microscope Model IX70 (20× magnification). The percentage of dead cells was estimated by calculating the number of Hoechst-stained nuclei relative to the total cell number in each field.

### Cell cycle analysis

Cells were plated in medium supplemented with 5% FBS and treated as indicated. After treatment the cells were trypsinized, washed once with PBS, and fixed in cold methanol for 15 minutes. Fixed cells were washed once with PBS and incubated at 4°C for 30 minutes. RNase A (0.05 mg/ml) and propidium iodide (0.05 mg/ml) were added and the stained cells were analyzed in a fluorescence-activated cell sorter (FACScan; Becton Dickinson, Franklin Lakes, NJ) within 30 minutes. Percentages of cells at different stages of the cell cycle were determined using the WinMDI 2.9 program.

### Clonogenic assay

HCT-116, DLD-1 and HELA cells were plated at a density of 60*10^3^, 50*10^3^, 75*10^3^ cells, respectively, onto 6-well plate, grown for 24 hr and then treated as indicated. After treatment, the cells were detached and replated on 10-cm plates (1:10, 1:400, 1:40 dilution, respectively). 5,7,10 days later, respectively, the cells were fixed with 0.1% acetic acid in PBS and then stained with 0.4% crystal violet in acetic acid. Total colonies area was calculated using the ImageJ program

### Lysate preparation and immunoprecipitation

After the indicated treatment, cells were lysed in solubilization buffer (50 mM HEPES pH 7.5, 150 mM NaCl, 10% glycerol, 1% Triton X-100, 1 mM EDTA pH 8, 1 mM EGTA pH 8, 1.5 mM MgCl_2_, 200 μM Na_3_VO_4_, 150 nM aprotinin, 1 μM leupeptin and 500 μM 4-(2-aminoethyl) benzenesulfonyl fluoride hydrochloride (Sigma). Lysates were cleared by centrifugation and a boiling gel sample buffer was added. Lysates were resolved by sodium dodecyl sulfate polyacrylamide gel electrophoresis through 10%‒12.5% polyacrylamide gels, and were electrophoretically transferred to nitrocellulose membranes. Membranes were blocked for 1 hour in TBST buffer (0.05 M Tris-HCl pH 7.5, 0.15 M NaCl, and 0.1% Tween 20) containing 6% milk, and then blotted with primary antibodies for 2 hours. Secondary antibody linked to horseradish peroxidase was then added for 1 hour. Immunoreactive bands were detected with the enhanced chemiluminescence reagent.

### Statistical analysis

All experiments were performed at least three times. Results are presented as means ± SD. Differences between means were assessed by the 1-tailed Student's *t*-test. Significance was assigned at p < 0.05.
